# Nonlinear Ride Height Control of Active Air Suspension System with Output Constraints and Time-Varying Disturbances

**DOI:** 10.3390/s21041539

**Published:** 2021-02-23

**Authors:** Rongchen Zhao, Wei Xie, Jin Zhao, Pak Kin Wong, Carlos Silvestre

**Affiliations:** 1 School of Mechanical and Electrical Engineering, Guizhou Normal University, Guizhou 550001, China; zhaorongchen@outlook.com; 2 Department of Electrical and Computer Engineering, University of Macau, Macau 999078, China; csilvestre@um.edu.mo; 3 Key Laboratory of Advanced Manufacturing Technology of the Ministry of Education, Guizhou University, Guizhou 550025, China; zhaoj@gzu.edu.cn; 4 Department of Electromechanical Engineering, University of Macau, Macau 999078, China; fstpkw@um.edu.mo

**Keywords:** nonlinear height control, active air suspension, output constraints, random road excitation, disturbance observer design

## Abstract

This paper addresses the problem of nonlinear height tracking control of an automobile active air suspension with the output state constraints and time-varying disturbances. The proposed control strategy guarantees that the ride height stays within a predefined range, and converges closely to an arbitrarily small neighborhood of the desired height, ensuring uniform ultimate boundedness. The designed nonlinear observer is able to compensate for the time-varying disturbances caused by external random road excitation and perturbations, achieving robust performance. Simulation results obtained from the co-simulation (AMESim-Matlab/Simulink) are given and analyzed, demonstrating the efficiency of the proposed control methodology.

## 1. Introduction

Active vehicle suspensions are effective ways to isolate or dissipate the vibration energies transferred from irregular road excitation to vehicle body [1,2,3]. With the development of automobile industry, the active suspension has demonstrated its capability in (1) improving ride comfort, i.e., reducing vehicle body acceleration, and (2) the safety performance constraint, such as suspension dynamic displacement, tire dynamic payload, and actuator input saturation [2,4,5,6]. As it is convenient to employ electronically-controlled active air suspension (AAS) systems to adjust the ride height by inflating and deflating the air spring, they have drawn attention from automobile manufactories (e.g., Tesla) and have been extensively utilized in commercial vehicles [7,8,9].

However, there are still many challenges in regulating the ride height motion of the vehicle body (with the AAS system) robustly and accurately under random road excitation. Moreover, the adjustment of ride height usually changes the stiffness and hysteresis, and generates perturbations in the AAS system [10,11]. In addition, because of the mechanical structure and travel limitations of the AAS, the ride height movement should always be constrained in a reliable range for safety performance [12,13,14]. Therefore, an appropriate ride height controller should be designed for the AAS systems in the presence of perturbations and output constraints.

Aiming to deal with the aforesaid problems, many results have been reported, such as robust $H_{\infty }$ control [15,16], sliding mode control (SMC) [11,17,18], fuzzy logic [19], neural network-based [20], and backstepping control techniques [14,21]. In [16], a robust $H_{\infty }$ controller for AAS systems was proposed, where the ride comfort and time domain hard constraints were considered. However, the model uncertainties are linearized by transformation of their utilized dynamic model [16]. As this model cannot capture the actual behavior of the AAS system, it could deteriorate the height tracking performance. Nonlinear controllers were proposed by employing SMC technique to handle the external random road excitation and perturbations in the AAS system [11,17,18]. However, the authors of [11] dealt with the time-varying disturbances by choosing high control gains for robustness resulting in unwanted oscillations. To cope with this drawback, an adaptive SMC scheme was proposed by using neural networks to increase SMC properties in [18]. Simulation results and Lyapunov-based stability proof were presented, demonstrating the proposed control method can stabilize displacement and speed of the suspension systems. Similarly, the backstepping control has been extensively investigated by employing fuzzy logic and neural networks for enhancing the control performance [19,20]. However, in order to make the approximation error arbitrarily small, the numbers of FLS rules or neurons should be increased, resulting in a heavy computational burden. Meanwhile, to our best knowledge, the numbers of the needed rules or neurons are difficult to be determined for keeping the estimation error bounded in a specific range. In [14,21], nonlinear backstepping-based height tracking controllers were designed, where some conservativeness was adopted in the control law to reduce the effects of time-varying disturbances.

In addition to the challenge raised from developing control strategy for handling disturbances, the output height constraint is also considered as a critical issue due to the mechanical structure limitation of the AAS system. Although the nonlinear ride height controllers based on the classic Quadratic Lyapunov function are presented to track predefined trajectories in the presence of perturbations and the height constraint are neglected for the ride height control applications with the AAS system [14,21]. By using the backstepping control approaches, the Barrier Lyapunov Functions (BLFs) have been developed and defined as control Lyapunov candidates for achieving the constrained objectives control [13,22,23,24]. In [22], the BLFs are employed in the controller design. Moreover, an asymmetric BLF is presented and employed in the constrained controller design to handle the external disturbances without violation of the output constraints [23]. In addition, the author of [13] proposed the constrained adaptive controller for damping force control by using the BLFs, improving ride comfort, and satisfying the performance constraints. However, the height motion control of AAS systems in the presence of output constraint has not been addressed yet.

Inspired by the above discussion, this paper presents a novel solution to address the height tracking control problem of nonlinear AAS system with output constraint and external time-varying disturbances. The novelties and contributions of this paper are summarized as follows.

- A nonlinear height tracking controller for the nonlinear AAS system is proposed, guaranteeing that (i) the output height always stays in a predefined range and (ii) uniform ultimate boundedness is achieved.
- A nonlinear disturbance observer is designed to compensate the time-varying disturbances caused by external random road excitation and perturbations in the AAS system.

With respect to the state-of-the art approaches, the main merits of the proposed constrained control strategy are as follows. In this research, unlike the linearized models used in [16], the mathematical model with the time-varying disturbances is employed to describe the perturbations in the AAS system. Unlike the disturbance rejection methods presented in [14,19,20,21], a time-varying disturbance observer is designed in this paper, guaranteeing that the estimation error is bounded by certain value. The designed disturbance observer can guarantee the estimate converges closely to zero. Moreover, we take the output constraints into consideration by using the BLFs in the backstepping controller design. By contrast, the output constraint was neglected in [11,17,19,20].

The remainder of this paper is organized as follows. In Section 2, the notation used throughout this paper is introduced. Section 3 presents a mathematical model of the AAS system and formulates the control problem. In Section 4, a nonlinear disturbance observer is designed, and a constrained ride height controller is proposed based on the backstepping control technique with BLFs. To validate the efficiency of proposed control strategy, co-simulation results are presented and analyzed in Section 5. At last, Section 6 summarizes the contents of this paper and describes the future work.

## 2. Notation

In this paper, $R^{n}$ denotes the n−dimensional euclidean space. A function *f* is of class $C^{n}$ if the derivatives $f^{{}^{\prime }}$, $f^{{}^{\prime \prime }}$, ..., $f^{n}$ exist and are continuous. For a vector $x\in R^{n}$, its norm is defined as $∥x∥=\sqrt{x^{T}x}$. $\int_{0}^{t}x\;d\tau$ denotes the integral of x, and τ denotes the integration variable. For the reader’s reference, Table 1 summarizes the main symbols and descriptions for the model, controller, and parameter estimators in the paper.

## 3. Problem Formulation

The objective of this section is to formulate the problem of nonlinear ride height tracking control with application to the AAS system in the presence of output constraints and the time-varying disturbances. We start by presenting the mathematical model of a quarter vehicle with AAS system. Then, the problem of constrained ride height tracking control is formulated with the maximum boundary value of vehicle ride height and the time-varying disturbances.

### 3.1. ASS Modeling

**Figure 1:**
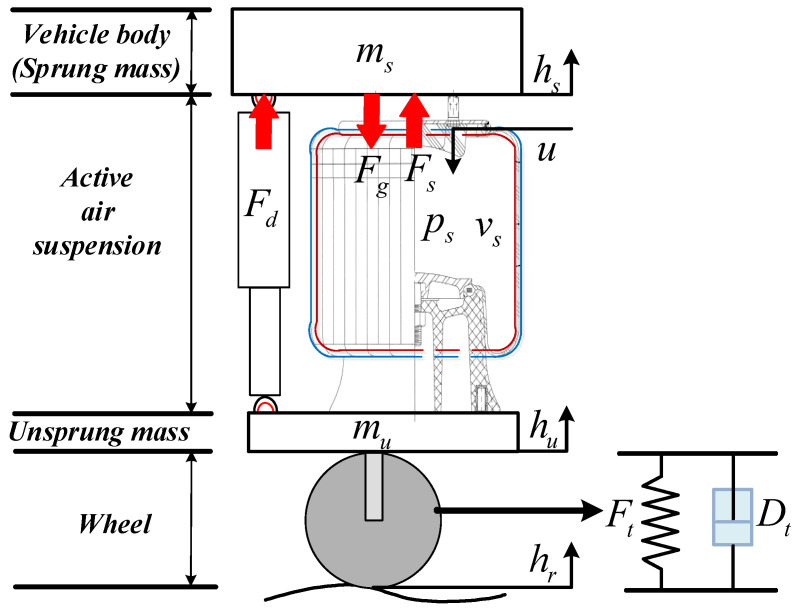
Schematic diagram of quarter vehicle with active air suspension (AAS).

In order to describe the dynamic characteristics, a mathematical model of a quarter vehicle with AAS is employed as a part of model-based height controller design for realizing the control objective successfully. The schematic diagram of a quarter vehicle with AAS is shown in Figure 1, and the dynamic equations of the sprung mass and unsprung mass are given by

(1) $$\begin{array}{cc} m_{s}{\ddot{h}}_{s} & =F_{s}-F_{d}-F_{g}\\ m_{u}{\ddot{h}}_{u} & =-F_{s}+F_{d}-F_{t}-D_{t} \end{array}$$

where $F_{g}=m_{s}g$ denotes the gravitational force; $m_{s}$ is the sprung mass of a quarter vehicle; $m_{u}$ is the unsprung mass, which denotes mass of the wheel assembly; and $F_{s}$ and $F_{d}$ represent the forces produced by the air spring and damper, respectively. $F_{t}$ and $D_{t}$ are the elasticity force and damping force of the tire, respectively. Forces produced by the nonlinear air spring, the linear damper, and the tire yield,

(2) $$\begin{array}{cc} F_{s} & ={\bar{A}}_{s}\left(p_{s}-p_{a}\right)\\ F_{d} & =b({\dot{h}}_{s}-{\dot{h}}_{u})\\ F_{t} & =k_{t}\left(h_{u}-h_{r}\right)\\ D_{t} & =b_{t}\left({\dot{h}}_{u}-{\dot{h}}_{r}\right) \end{array}$$

where $p_{a}$ is the atmospheric pressure, $s_{p}=10^{5}$, and ${\bar{A}}_{s}=A_{s}s_{p}$ is the effective area of the adjustable air spring. $h_{r}$ denotes the random road excitation, and $h_{u}$ and $h_{s}$ are the displacements of the unsprung mass and the sprung mass of the quarter vehicle, respectively. *b* is the damping coefficient, $k_{t}$ and $b_{t}$ are the stiffness and damping coefficient of the tire, respectively.

Due to the perturbations in the AAS system, the time-varying disturbances should be considered in the employed model for ride height control. From (1), the quarter vehicle model with the AAS can be then expressed in a compact state-space form as

(3) $$\begin{array}{cc} {\dot{h}}_{1} & =h_{2}\\ {\dot{h}}_{2} & =m_{s}^{-1}\left({\bar{A}}_{s}\left(h_{3}-p_{atm}\right)-b\left(h_{2}-{\dot{z}}_{u}\right)-m_{s}g\right)\\ {\dot{h}}_{3} & =u-\gamma A_{s}h_{2}h_{3}v_{s}^{-1}+\dot{Q}\left(\gamma -1\right){\left(s_{p}v_{s}\right)}^{-1}+d\left(t\right) \end{array}$$

where $h_{1}=h_{s},\;h_{2}={\dot{h}}_{s},\;h_{3}=p_{s}s_{p}^{-1}$. $v_{s}$ represents the air volume; $\dot{Q}=h_{t}A_{heat}\left(T_{e}-T_{as}\right)$ is the heat transfer rate between the inner and the outer sides of the control volume, in which $h_{t}$ is the heat transfer coefficient; $A_{heat}$ represents the area of the heat transfer; $T_{e}$ and $T_{as}$ are temperatures of the outer and the inner sides of the control volume, respectively; and *u* is viewed as the control input for inflating and deflating the adjustable air spring. The time-varying disturbances are denoted by d(t) with the following assumption:

**Assumption** **1.** *Disturbances d(t) are unknown, time-varying and satisfy*

$$\left|d\left(t\right)\right|\leq d_{max},\;\;\;\left|\dot{d}\left(t\right)\right|\leq \bar{d}$$

*where $d_{max}$ and $\bar{d}$ are known positive numbers.*

### 3.2. Output Constraint and Barrier Lyapunov Function

In practice, because of the structure limitation, the output height of the AAS system should be constrained by $h_{\max}$, which denotes the maximum ride height of the AAS. Inspired by the work in [24], the following BLF is used.

(4) $$\begin{array}{c} V_{b}=\frac{1}{2}\Upsilon^{2}\left(z_{1}\right) \end{array}$$

where

(5) $$\begin{array}{c} \Upsilon \left(z_{1}\right)=\frac{\mu^{2}z_{1}}{\mu^{2}-z_{1}^{2}} \end{array}$$

where $z_{1}=h_{1}-h_{d}$ is the velocity tracking error, $h_{d}$ denotes the desired height under the assumption that $|h_{d}|<\bar{h}<h_{\max}$, and $\mu =h_{\max}-\bar{h}$. To facilitate the analysis, we formulate a simple lemma, given as

**Lemma** **1.** *For any two nonzero scalars x∈R,y∈R, if $|x|<x_{\max},\;\left|y\right|<y_{\max},\;x_{\max}>y_{\max}>0$, then we have*

(6) $$\begin{array}{c} |x|-|y|\leq |x-y|. \end{array}$$

Furthermore, based on Equation (6), if we have $|x-y|<x_{\max}-y_{\max},\;\left|y\right|<y_{\max},x_{\max}>y_{\max}>0$, we can obtain $|x|<x_{\max}.$

**Remark** **1.** *The BLF $V_{b}$ is positive definite and $C^{1}$ continuous for $|z_{1}|<\mu$.*

**Remark** **2.** *If there is no constraint on $h_{1}$, that is, $h_{\max}\to +\infty$, the BLF becomes*

(7) $$\begin{array}{c} V_{b}=\frac{1}{2}z_{1}^{2} \end{array}$$

*which is a quadratic Lyapunov function.*

### 3.3. Problem Statement

For AAS systems, the following control objectives should be considered in the ride height controller design.

- The proposed ride height controller can guarantee the accurate trajectory tracking performance in the presence of time-varying disturbances.
- Due to the mechanical structure and travel limitation of the AAS, the dynamic height should be restrained within its allowable maximum value, which is expressed as $|h_{s}|<h_{\max}$.

## 4. Nonlinear Backstepping Controller Synthesis

In this section, the control objective is to design the virtual control input *u* for the ASS that ensures convergence of the ride height to an arbitrarily small neighborhood of the desired height without violating the requirement of output constraint $|h_{1}|<h_{\max}$. A disturbance observer $\hat{d}\left(t\right)$ is first designed to estimates d(t), and then a constrained controller is designed based on the backstepping technique by using the BLF. Details are given in the sequel.

### 4.1. Disturbance Observer Design

To design the disturbance observer for d(t), we take a clue from the work in [25] and define two auxiliary terms as

(8) $$\begin{array}{cc} \xi & =d\left(t\right)-\phi \left(h_{3}\right),\\ \hat{\xi } & =\hat{d}\left(t\right)-\phi \left(h_{3}\right), \end{array}$$

where $\phi \left(h_{3}\right)=\lambda_{d}h_{3}$, $\lambda_{d}$ is a positive estimation gain, and $\hat{d}\left(t\right)$ is the estimation of d(t). From (8), we have

(9) $$\begin{array}{c} \hat{d}\left(t\right)=\hat{\xi }+\phi \left(h_{3}\right) \end{array}$$

and the estimation error is

(10) $$\begin{array}{c} d_{e}=\xi -\hat{\xi }. \end{array}$$

Computing the time derivative of ξ, we obtain

(11) $$\begin{array}{cc} \dot{\xi } & =\dot{d}\left(t\right)-\frac{\partial \phi (h_{3})}{\partial h_{3}}\left[u-\gamma A_{s}h_{2}h_{3}v_{s}^{-1}+\dot{Q}\left(\gamma -1\right){\left(s_{p}v_{s}\right)}^{-1}+d\left(t\right)\right]. \end{array}$$

Then, we introduce the time derivative of the estimated $\hat{\xi }$, given as

(12) $$\begin{array}{cc} \dot{\hat{\xi }} & =-\frac{\partial \phi (h_{3})}{\partial h_{3}}\left[u-\gamma A_{s}h_{2}h_{3}v_{s}^{-1}+\dot{Q}\left(\gamma -1\right){\left(s_{p}v_{s}\right)}^{-1}+\hat{d}\left(t\right)\right], \end{array}$$

leading to

(13) $$\begin{array}{cc} {\dot{d}}_{e} & =\dot{\xi }-\dot{\hat{\xi }}\\ & =\dot{d}\left(t\right)-\lambda_{d}d_{e}. \end{array}$$

The main result is summarized in the following Lemma.

**Lemma** **2.** *Through the use of designed disturbance observer* (9)*, the estimate $|d_{e}\left(t\right)|$ exponentially converges to the circle centered at the origin with radius $d_{e}\left(t\right){\left(4\varepsilon \left(\lambda_{d}-\varepsilon \right)\right)}^{-\frac{1}{2}}$, which can be made arbitrarily small by increasing the estimation gain $\lambda_{d}$, where ε is a positive constant.*

**Proof.**  We start the proof by defining a Lyapunov candidate function, given as

(14) $$\begin{array}{c} V_{d}\left(t\right)=\frac{1}{2}d_{e}{\left(t\right)}^{2}. \end{array}$$

Computing the time derivative of $V_{d}\left(t\right)$, we have

(15) $$\begin{array}{cc} {\dot{V}}_{d}\left(t\right) & =-\lambda_{d}d_{e}{\left(t\right)}^{2}+d_{e}\left(t\right)\dot{d}\left(t\right)\\ & \leq -2\left(\lambda_{d}-\varepsilon \right)V_{d}\left(t\right)+{\bar{d}}^{2}{\left(4\varepsilon \right)}^{-1}, \end{array}$$

where $\lambda_{d}>\varepsilon$. Solving (15), we obtain

(16) $$\begin{array}{c} V_{d}\left(t\right)\leq e^{-2(\lambda_{d}-\varepsilon )}V_{d}\left(0\right)+{\bar{d}}^{2}{\left(8\varepsilon \left(\lambda_{d}-\varepsilon \right)\right)}^{-1}. \end{array}$$

From here we can conclude that $V_{d}$ converges to a circle of radius ${\bar{d}}^{2}{\left(8\varepsilon \left(\lambda_{d}-\varepsilon \right)\right)}^{-1}$. It follows that $|d_{e}\left(t\right)|$ converges to a circle of radius $\bar{d}{\left(4\varepsilon \left(\lambda_{d}-\varepsilon \right)\right)}^{-\frac{1}{2}}$, which can be made arbitrarily small by increasing $\lambda_{d}$. □

### 4.2. Constrained Controller Design

Let the desired height $h_{d}$ be a curve of class at least $C^{3}$, with all its time derivatives bounded. In order to address the constrained height tracking problem, we consider (4) as an initial Lyapunov function candidate given by

(17) $$\begin{array}{c} V_{1}=V_{b}=\frac{1}{2}\Upsilon {\left(z_{1}\right)}^{2}, \end{array}$$

whose time derivative yields

(18) $$\begin{array}{c} {\dot{V}}_{1}=\Upsilon \left(z_{1}\right)\dot{\Upsilon }\left(z_{1}\right), \end{array}$$

where

(19) $$\begin{array}{c} \dot{\Upsilon }\left(z_{1}\right)=\frac{2\mu^{2}z_{1}^{2}}{{\left(\mu^{2}-z_{1}^{2}\right)}^{2}}{\dot{z}}_{1}+\frac{\mu^{2}}{\mu^{2}-z_{1}^{2}}{\dot{z}}_{1}, \end{array}$$

For the sake of simplicity, we define $\delta_{1}$ and $\delta_{2}$ as

(20) $$\begin{array}{c} \delta_{1}=\frac{2\mu^{2}z_{1}^{2}}{{\left(\mu^{2}-z_{1}^{2}\right)}^{2}},\delta_{2}=\frac{\mu^{2}}{\mu^{2}-z_{1}^{2}}, \end{array}$$

then, Equation (18) can be rewritten as

(21) $$\begin{array}{c} {\dot{V}}_{1}=\Upsilon \left(z_{1}\right)\left(\delta_{1}{\dot{z}}_{1}+\delta_{2}{\dot{z}}_{1}\right). \end{array}$$

Isolating a negative definite term in $\Upsilon (z_{1})$ and rearranging the terms of ${\dot{V}}_{1}$, we get

(22) $$\begin{array}{c} {\dot{V}}_{1}=-W_{1}\left(z_{1}\right)+\Upsilon \left(z_{1}\right)\left(\delta_{1}{\dot{z}}_{1}+\delta_{2}{\dot{z}}_{1}+k_{1}\Upsilon \left(z_{1}\right)\right). \end{array}$$

where $W_{1}\left(z_{1}\right)=k_{1}\Upsilon {\left(z_{1}\right)}^{2}$, and $k_{1}$ is a positive number. Following the backstepping technique, we define the new error $z_{2}$ as

(23) $$\begin{array}{c} z_{2}=\delta_{1}{\dot{z}}_{1}+\delta_{2}{\dot{z}}_{1}+k_{1}\Upsilon \left(z_{1}\right), \end{array}$$

and rewriting (22), we have

(24) $$\begin{array}{c} {\dot{V}}_{1}=-W_{1}\left(z_{1}\right)+\Upsilon \left(z_{1}\right)z_{2}. \end{array}$$

Constructing a new Lyapunov function candidate by incorporating $z_{2}$, we obtain

(25) $$\begin{array}{c} V_{2}=\frac{1}{2}\Upsilon {\left(z_{1}\right)}^{2}+\frac{1}{2}z_{2}^{2}, \end{array}$$

with time derivative

(26) $$\begin{array}{cc} {\dot{V}}_{2} & =-W_{2}\left(z_{1},\;z_{2}\right)+z_{2}(\left(m_{s}^{-1}{\bar{A}}_{s}\left(h_{3}-p_{atm}\right)-m_{s}^{-1}b\left(h_{2}-{\dot{z}}_{u}\right)-g-{\ddot{h}}_{d}\right)\left(\delta_{1}+\delta_{2}\right)\\ & +\Upsilon \left(z_{1}\right)+\left({\dot{\delta }}_{1}+{\dot{\delta }}_{2}\right){\dot{z}}_{1}+k_{1}\dot{\Upsilon }\left(z_{1}\right)+k_{2}z_{2}), \end{array}$$

where $W_{2}\left(z_{1},\;z_{2}\right)=W_{1}\left(z_{1}\right)+k_{2}z_{2}^{2}$, $k_{2}$ is a positive number,

(27) $$\begin{array}{cc} {\dot{\delta }}_{1} & =\frac{4\mu^{2}z_{1}{\dot{z}}_{1}}{{\left(\mu^{2}-z_{1}^{2}\right)}^{2}}+\frac{8\mu^{2}z_{1}^{3}{\dot{z}}_{1}}{{\left(\mu^{2}-z_{1}^{2}\right)}^{3}},\;{\dot{\delta }}_{2}=\frac{2\mu^{2}z_{1}{\dot{z}}_{1}}{{\left(\mu^{2}-z_{1}^{2}\right)}^{2}}. \end{array}$$

Furthermore, we can rewrite (26) as

(28) $$\begin{array}{cc} {\dot{V}}_{2} & =-W_{2}\left(z_{1},\;z_{1}\right)+z_{2}\left(\delta_{1}+\delta_{2}\right)[m_{s}^{-1}{\bar{A}}_{s}\left(h_{3}-p_{atm}\right)-m_{s}^{-1}b\left(h_{2}-{\dot{z}}_{u}\right)-g-{\ddot{h}}_{d}\\ & +\frac{\Upsilon (z_{1})}{\delta_{1}+\delta_{2}}+\left(\frac{\dot{\delta_{1}}+\dot{\delta_{2}}}{\delta_{1}+\delta_{2}}+k_{1}\right){\dot{z}}_{1}+\frac{k_{2}z_{2}}{\delta_{1}+\delta_{2}}]. \end{array}$$

Continuing with the backstepping procedure, we define the last error term as

(29) $$\begin{array}{cc} z_{3} & =m_{s}^{-1}{\bar{A}}_{s}\left(h_{3}-p_{atm}\right)-m_{s}^{-1}b\left(h_{2}-{\dot{z}}_{u}\right)-g-{\ddot{h}}_{d}+\frac{\Upsilon (z_{1})}{\delta_{1}+\delta_{2}}\\ & +\left(\frac{\dot{\delta_{1}}+\dot{\delta_{2}}}{\delta_{1}+\delta_{2}}+k_{1}\right){\dot{z}}_{1}+\frac{k_{2}z_{2}}{\delta_{1}+\delta_{2}}, \end{array}$$

and augment the Lyapunov function candidate as

(30) $$\begin{array}{c} V_{3}=V_{2}+\frac{1}{2}z_{3}^{2}. \end{array}$$

The closed-loop time derivative is then

(31) $$\begin{array}{cc} {\dot{V}}_{3} & =-W_{3}\left(z_{1},\;z_{2},\;z_{3}\right)+z_{3}[m_{s}^{-1}{\bar{A}}_{s}{\dot{h}}_{3}-m_{s}^{-1}b\left(h_{2}-{\dot{z}}_{u}\right)-h_{d}^{\left(3\right)}\\ & +\left(\frac{{\dot{\delta }}_{1}+{\dot{\delta }}_{2}}{\delta_{1}+\delta_{2}}+k_{1}+k_{2}+1\right){\ddot{z}}_{1}+(\frac{{\ddot{\delta }}_{1}+{\ddot{\delta }}_{2}}{\delta_{1}+\delta_{2}}-\frac{{\dot{\delta }}_{1}+{\dot{\delta }}_{2}}{{\left(\delta_{1}+\delta_{2}\right)}^{2}}\\ & +\frac{k_{1}k_{2}}{\delta_{1}+\delta_{2}}){\dot{z}}_{1}+\frac{{\dot{\delta }}_{1}+{\dot{\delta }}_{2}}{{\left(\delta_{1}+\delta_{2}\right)}^{2}}k_{1}k_{2}\Upsilon \left(z_{1}\right)\\ & +\left(\delta_{1}+\delta_{2}\right)z_{2}+k_{3}z_{3}]. \end{array}$$

where

(32) $$\begin{array}{cc} {\ddot{\delta }}_{1} & =\frac{4\mu^{2}{\dot{z}}_{1}^{2}}{{\left(\mu^{2}-z_{1}^{2}\right)}^{2}}+\frac{40\mu^{2}z_{1}^{2}{\dot{z}}_{1}^{2}}{{\left(\mu^{2}-z_{1}^{2}\right)}^{3}}+\frac{48\mu^{2}z_{1}^{4}{\dot{z}}_{1}^{2}}{{\left(\mu^{2}-z_{1}^{2}\right)}^{4}}+\frac{4\mu^{2}z_{1}{\ddot{z}}_{1}}{{\left(\mu^{2}-z_{1}^{2}\right)}^{2}}+\frac{8\mu^{2}z_{1}^{3}{\ddot{z}}_{1}}{{\left(\mu^{2}-z_{1}^{2}\right)}^{3}},\\ {\ddot{\delta }}_{1} & =\frac{2\mu^{2}{\dot{z}}_{1}^{2}}{{\left(\mu^{2}-z_{1}^{2}\right)}^{2}}+\frac{8\mu^{2}z_{1}^{2}{\dot{z}}_{1}^{2}}{{\left(\mu^{2}-z_{1}^{2}\right)}^{3}}+\frac{2\mu^{2}z_{1}{\ddot{z}}_{1}}{{\left(\mu^{2}-z_{1}^{2}\right)}^{2}}, \end{array}$$

and $W_{3}\left(z_{1},\;z_{2},\;z_{3}\right)=W_{2}\left(z_{1},\;z_{2}\right)+k_{3}z_{3}^{2}$, and $k_{3}$ is a positive number

Here, note that the time derivative of ${\dot{V}}_{3}$ is dependent on the disturbances d(t) through the dependency of ${\dot{h}}_{3}$ in these quantities. In order to exploit the dependency of ${\dot{h}}_{3}$ in the uncertain quantities, the time derivative ${\dot{h}}_{3}$ can be expressed as

(33) $$\begin{array}{cc} {\dot{h}}_{3} & =u-\gamma A_{s}h_{2}h_{3}v_{s}^{-1}+\dot{Q}\left(\gamma -1\right){\left(s_{p}v_{s}\right)}^{-1}+\hat{d}\left(t\right)+d_{e}\left(t\right), \end{array}$$

where $d_{e}\left(t\right)$ is the estimation error. We now establish the final Lyapunov function candidate by adding the terms of disturbance estimate error to $V_{3}$ as follows,

(34) $$\begin{array}{cc} V_{3b} & ={\dot{V}}_{3}+\frac{1}{2}d_{e}{\left(t\right)}^{2}. \end{array}$$

Computing the time derivative of $V_{3b}$, we obtain

(35) $$\begin{array}{cc} {\dot{V}}_{3b} & =-W_{3}\left(z_{1},\;z_{2},\;z_{3}\right)+z_{3}[M+m_{s}^{-1}{\bar{A}}_{s}(u-\gamma A_{s}h_{2}h_{3}v_{s}^{-1}+\dot{Q}\left(\gamma -1\right){\left(s_{p}v_{s}\right)}^{-1}\\ & +\hat{d}\left(t\right))-m_{s}^{-1}b\left(h_{2}-{\dot{z}}_{u}\right)-h_{d}^{\left(3\right)}]+z_{3}m_{s}^{-1}{\bar{A}}_{s}d_{e}\left(t\right)+d_{e}\left(t\right)\left(\dot{d}\left(t\right)-d_{e}\left(t\right)\right). \end{array}$$

where

(36) $$\begin{array}{cc} M & =\left(\frac{{\dot{\delta }}_{1}+{\dot{\delta }}_{2}}{\delta_{1}+\delta_{2}}+k_{1}+k_{2}+1\right){\ddot{z}}_{1}+\left(\frac{{\ddot{\delta }}_{1}+{\ddot{\delta }}_{2}}{\delta_{1}+\delta_{2}}-\frac{{\dot{\delta }}_{1}+{\dot{\delta }}_{2}}{{\left(\delta_{1}+\delta_{2}\right)}^{2}}+\frac{k_{1}k_{2}}{\delta_{1}+\delta_{2}}\right){\dot{z}}_{1}\\ & +\frac{{\dot{\delta }}_{1}+{\dot{\delta }}_{2}}{{\left(\delta_{1}+\delta_{2}\right)}^{2}}k_{1}k_{2}\Upsilon \left(z_{1}\right)+\left(\delta_{1}+\delta_{2}\right)z_{2}+k_{3}z_{3}. \end{array}$$

Here, we notice that apart from the time derivative of disturbances $\dot{d}\left(t\right)$ and estimated error $d_{e}\left(t\right)$, ${\dot{V}}_{3b}$ is also dependent on the $z_{1},\;z_{2},\;z_{3}$. To cancel the dependency of ${\dot{V}}_{3b}$ on $z_{1},\;z_{2},\;z_{3}$ in (35), the virtual control law *u* is chosen as

(37) $$\begin{array}{cc} u & ={\bar{A}}_{s}^{-1}m_{s}\left(-M+h_{d}^{\left(3\right)}\right)+{\bar{A}}_{s}^{-1}b\left({\dot{h}}_{2}+{\ddot{z}}_{u}\right)+\gamma A_{s}h_{2}h_{3}v_{s}^{-1}\\ & -\dot{Q}\left(\gamma -1\right){\left(s_{p}v_{s}\right)}^{-1}-\hat{d}\left(t\right), \end{array}$$

Substituting (33) and (37) into (35), in closed-loop, we have

(38) $$\begin{array}{cc} {\dot{V}}_{3b} & =-k_{1}\Upsilon {\left(z_{1}\right)}^{2}-k_{2}z_{2}^{2}-k_{3}z_{3}^{2}-\lambda_{d}d_{e}{\left(t\right)}^{2}+z_{3}m_{s}^{-1}{\bar{A}}_{s}d_{e}\left(t\right)+\dot{d}\left(t\right)d_{e}\left(t\right). \end{array}$$

The main result of this paper is summarized in the following theorem.

**Theorem** **1.** *Let $h_{d}\in C^{3}$ in* (5) *be the desired height whose time derivatives are bounded, and $|z_{1}\left(0\right)|<\mu$. By considering the designed time-varying disturbance observer* (9) *and input* (37)*, the errors $z={\left[\Upsilon \left(z_{1}\right),\;z_{2},\;z_{3},\;d_{e}\right]}^{T}$ converge to an arbitrarily small neighborhood of zero, achieving uniform ultimate boundedness without violating the output constraint.*

**Proof.**  Let us go back to (38) and apply Young’s inequality, we have

(39) $$\begin{array}{c} {\dot{V}}_{3b}\leq -k_{1}\Upsilon {\left(z_{1}\right)}^{2}-k_{2}z_{2}^{2}-\left(k_{3}-\frac{m_{s}^{-1}{\bar{A}}_{s}}{4}\right)z_{3}^{2}-\left(\lambda_{d}-m_{s}^{-1}{\bar{A}}_{s}-1\right)d_{e}{\left(t\right)}^{2}+\frac{d_{max}^{2}}{4} \end{array}$$

where $k_{3}$ are chosen such that $k_{3}>m_{s}^{-1}{\bar{A}}_{s}/4$, $\lambda_{d}>1+m_{s}^{-1}{\bar{A}}_{s}$. Setting $z={\left[\Upsilon \left(z_{1}\right),\;z_{2},\;z_{3},\;d_{e}\right]}^{T}$, ${\dot{V}}_{3b}$ can be further written as

(40) $$\begin{array}{cc} {\dot{V}}_{3b} & \leq -k_{\min}{\left||z|\right|}^{2}+\frac{d_{max}^{2}}{4}\\ \; & =-k_{\min}\left({\left||z|\right|}^{2}-\frac{d_{max}^{2}}{4k_{\min}}\right) \end{array}$$

which is negative definite for

$$||z||>\sqrt{\frac{d_{max}^{2}}{4k_{\min}}}+\epsilon$$

where ϵ is an arbitrarily small positive constant. It follows that ||z|| is ultimately bounded by

(41) $$\begin{array}{c} z_{\max}=\sqrt{\frac{d_{max}^{2}}{4k_{\min}}}+\epsilon \end{array}$$

which can be made arbitrarily small by increasing the control gains, $k_{1},\;k_{2},\;k_{3}$ and $\lambda_{d}$. Consequently, global uniform ultimate boundedness is achieved. Notice that $|\Upsilon \left(z_{1}\right)\left|>\right|z_{1}|$, therefore, bounded $\left|\Upsilon (z_{1})\right|$ leads to bounded $|z_{1}|$. Moreover, it is important to point out that if the output constraint is violated, $\left|\Upsilon (z_{1})\right|$ will be infinity. However, as we established above, for $|z_{1}\left(0\right)|<\mu$, the error ∥z∥ will converge to a bounded value instead of infinity, from which we can conclude that the output constraint is guaranteed. □

**Remark** **3.** *From Theorem 1, we know that larger $k_{1},\;k_{2},\;k_{3},\;\lambda_{d}$ would lead to smaller ultimate error. However, larger gains could also cause unwanted oscillation. Consequently, we cannot choose them arbitrarily large. In summary, we need to find a trade-off between the amplitude of oscillation and tracking accuracy.*

## 5. Simulation Verification

**Figure 2:**
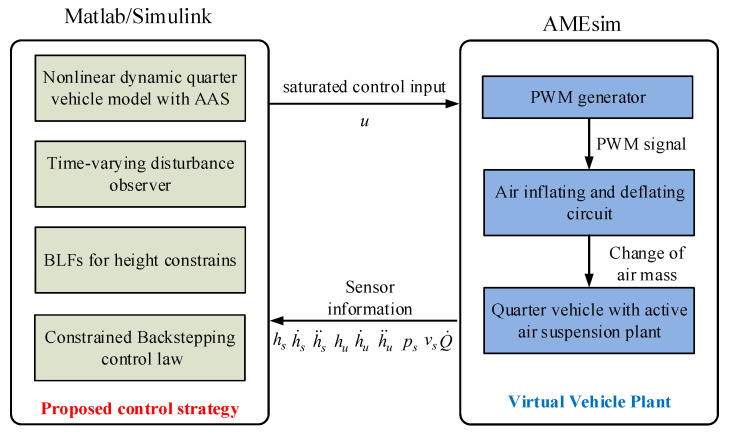
Co-simulation block diagram.

In order to verify the performance of proposed controller, a co-simulation is conducted in this section by combining the virtual plant of quarter vehicle with the AAS system in AMEsim software with the proposed controller in Matlab/Simulink to regulate the sprung height by inflating and deflating the air spring. The control block diagram of co-simulation is displayed in Figure 2. Unlike the mathematical model of the controller implemented in Matlab/Simulink, the AMESim-based quarter vehicle plant is established based on the actual pneumatic system configuration so that it is closer to the actual pneumatic system.

### 5.1. Simulation Conditions

The desired height $h_{d}$ is a sine trajectory, given by

(42) $$h_{d}=0.015\sin\left(\omega t\right),$$

where ω=0.5 (rad/s) . The time-varying disturbances are chosen as

(43) d(t)=sin(π/2t)sin(πt)+10sin(πt)cos(2πt)+ϑ(t),

where ϑ(t) is a class of band-limited white noise. The road excitation $h_{u}$ is set as the class C of ISO profile with a driving speed of $50\;(\mathrm{km}\cdot h^{-1})$, whose graphic representation is shown in Figure 2. The main parameters used in the co-simulation are given in Table 2, where the control gains are chosen through a trial–error process.

### 5.2. Simulation Results and Analysis

**Figure 3:**
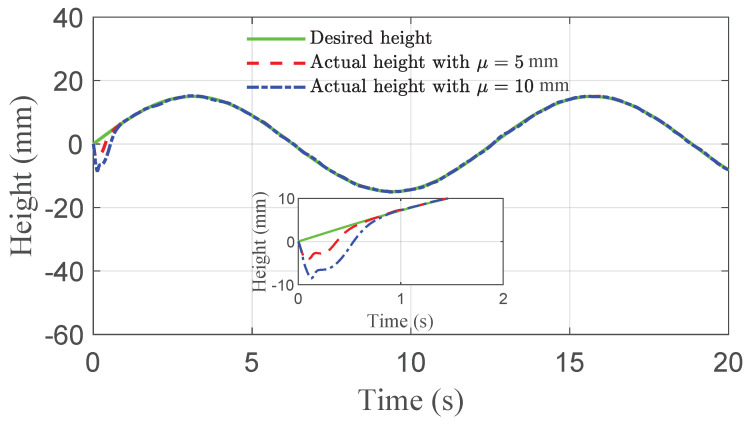
Height tracking performance of quarter vehicle with AAS in co-simulation.

**Figure 4:**
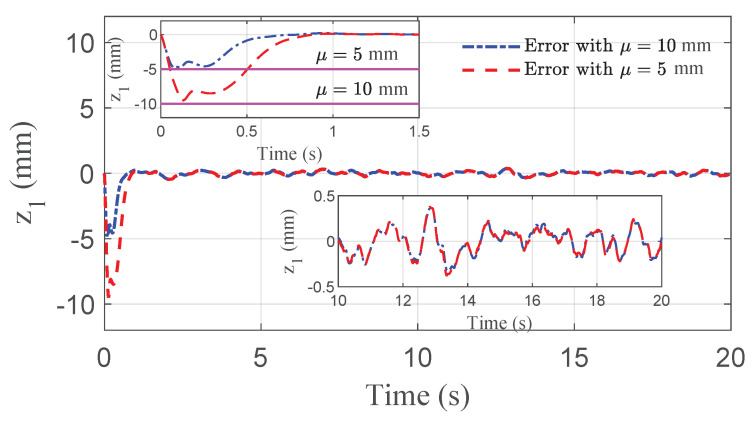
Height tracking error of quarter vehicle with AAS in co-simulation, where $|z_{1}|$ always stays within its corresponding bound.

**Figure 5:**
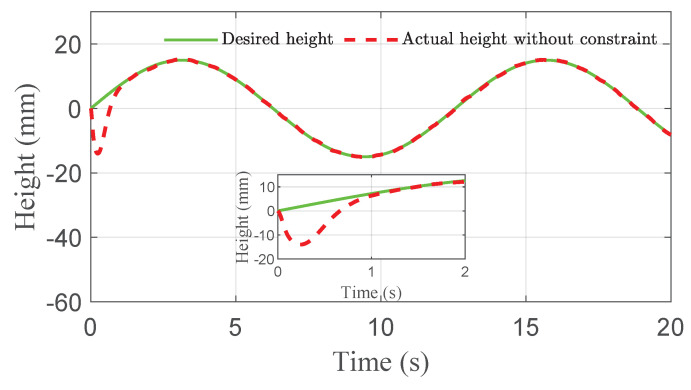
Height comparison of quarter vehicle with AAS in co-simulation.

**Figure 6:**
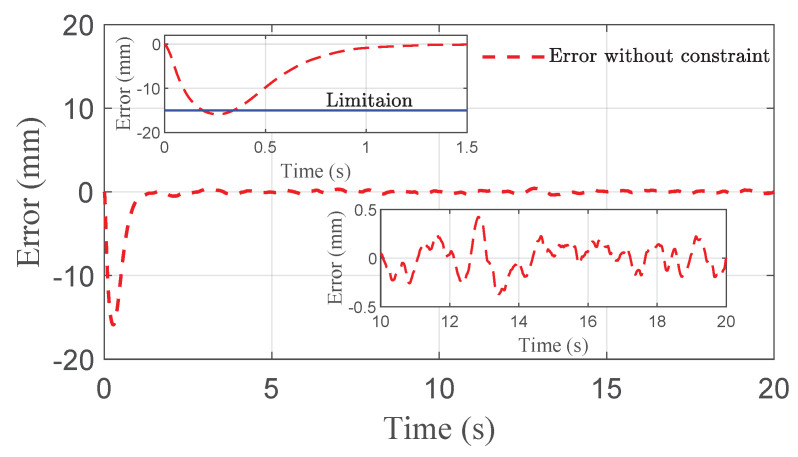
Error comparison of quarter vehicle with AAS in co-simulation, where $|z_{1}|$ exceeds its allowable maximum value.

**Figure 7:**
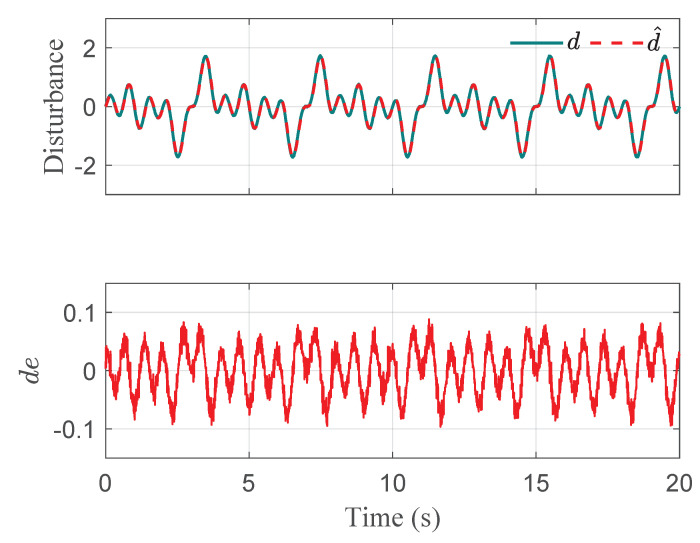
Disturbance estimation of quarter vehicle with AAS in co-simulation.

**Figure 8:**
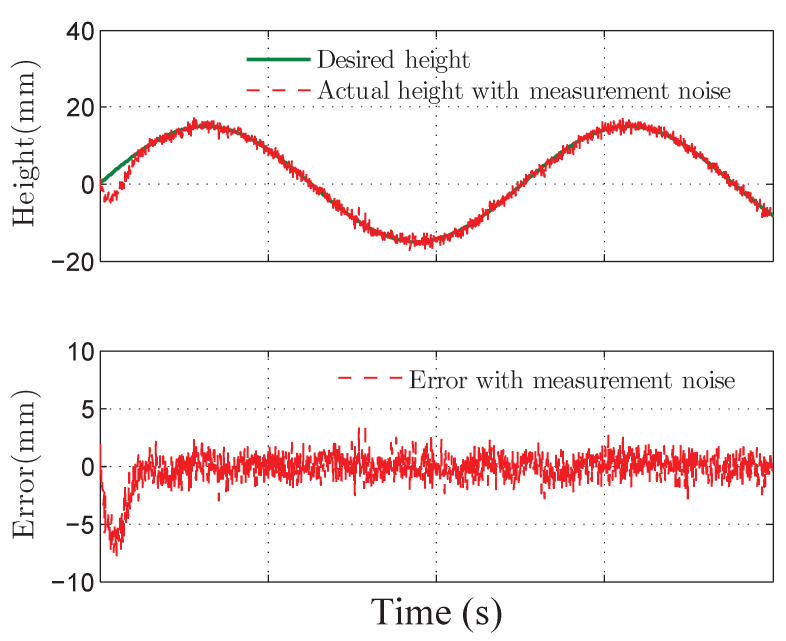
Tracking height and error of quarter vehicle with measurement noise in co-simulation.

The co-simulation results of the proposed control strategy for ride height control with the AAS system are displayed in Figure 3, Figure 4, Figure 5, Figure 6 and Figure 7. As shown in Figure 3, the height of vehicle sprung mass with the proposed controller can track the desired height within 1s. Moreover, the tracking errors, with μ=10mm and μ=5mm, all stay within the predefined range of ±10mm and ±5mm as shown in Figure 4. Moreover, compared with the proposed controller without considering the output state constraint (i.e., choosing $\mu_{\max}$ very large), although the height of vehicle sprung mass is able to track the desired value as displayed in Figure 5, the height tracking error exceeds its allowable maximum value that results probably in a poor performance or even insecurity as illustrated in Figure 6. Furthermore, the time-varying disturbances d(t) could be estimated by the developed nonlinear disturbance observer and $\hat{d}\left(t\right)$ can also be kept within the range of ±0.1 as depicted in Figure 7. It means that the designed disturbance observer is effective. Additionally, in order to simulate the real operating conditions, white noise is considered during the height measurement procedure. As Figure 8 displayed, the proposed controller still can track the desired height under the presence of measurement noise.

### 5.3. Comparison of Simulation Results

**Figure 9:**
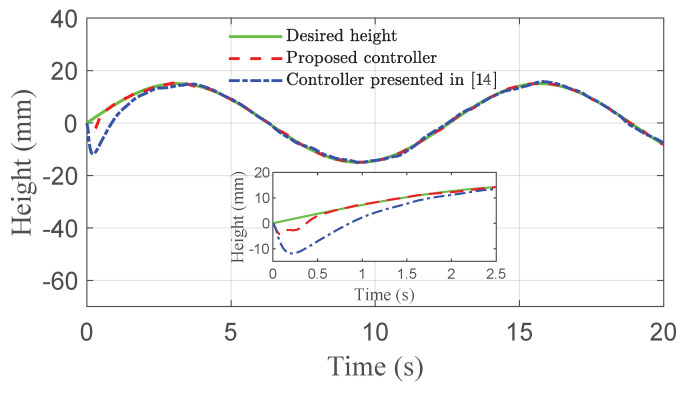
Height comparison of quarter vehicle with AAS in co-simulation.

**Figure 10:**
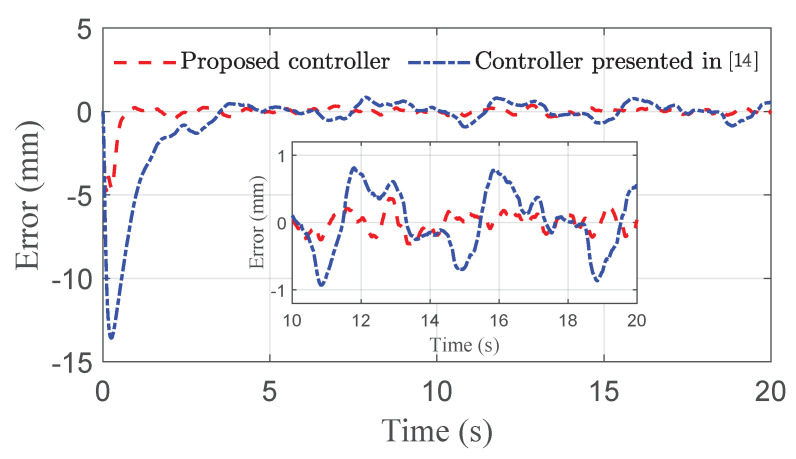
Error comparison of quarter vehicle with AAS in co-simulation.

**Figure 11:**
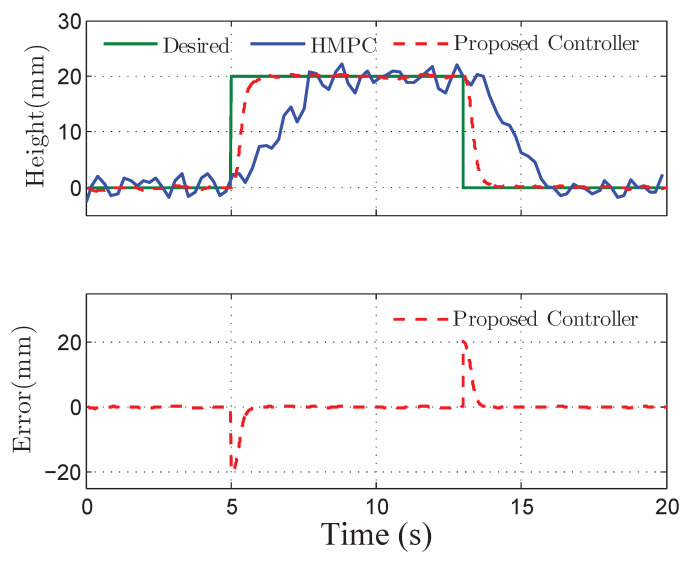
Tracking performance comparison of quarter vehicle with hybrid model predictive controller (HMPC) presented in [7].

In order to further demonstrate the benefits of the considerations of output state constraint and time-varying disturbances, Figure 9, Figure 10 and Figure 11 also show the simulation results of ride height with nonlinear robust controller [14] and hybrid model predictive controller (HMPC) [7] under the same simulation parameters, disturbances, and road excitation. As demonstrated in Figure 9, the height of vehicle sprung mass reaches the target value within 1s, which is much shorter than 4s obtained for the controller presented in [14]. Meanwhile, during the time from 10s to 20s, the steady-state error achieved by the robust controller presented in [14] is bounded by 0.8mm, which is larger than the bound of 0.5mm obtained with the proposed controller, as depicted in Figure 10. Moreover, the proposed controller can track the desired height during both leveling up and lowering down processes so that the height of vehicle sprung mass reaches the target height as illustrated in Figure 11. The desired height used in the test is presented in [7]. The simulation results in Figure 10 and Figure 11, and the performance comparison in Table 3 and Table 4 indicates that the proposed control technique is more effective than the robust controller presented in [14] and the HMPC presented in [7].

## 6. Conclusions

This paper presents a solution to the task of vehicle height tracking for an electronically controlled AAS system. By employing the BLF-based backstepping technique, a novel constrained control strategy is proposed to drive the vehicle height to the neighborhood of preset desired values in the presence of output state constrains and perturbations in the AAS system, achieving uniform ultimate boundedness. To realize the robust performance, a nonlinear disturbance observer is introduced in the adaptive control law to compensate for the time-varying disturbances caused the external random road excitation and perturbations, achieving robust performance. Co-simulation results illustrate that the proposed control strategy is effective, robust, and superior to other recent techniques. With respect to our future research, it includes (i) designing a robust height tracking controller for a full-car model with the AAS system, and (ii) developing a noise filter and delay compensator for the system so as to improve the closed-loop performance in real applications.

## Figures and Tables

**Table 1:** Symbols and their descriptions.

Symbol	Description	Symbol	Description
$h_{s}\;\left(m\right)$	height of vehicle sprung mass	m(kg)	sprung mass of quarter vehicle
$h_{u}\;\left(m\right)$	unsprung mass displacement	$m_{u}\;\left(\mathrm{kg}\right)$	unsprung mass of quarter vehicle
$h_{d}\;\left(m\right)$	desired height	${\dot{m}}_{des}\;\left(g\right)$	desired change of air mass for air spring
$h_{r}\;\left(m\right)$	road excitation	${\bar{A}}_{s}\;\left(mm^{2}\right)$	area of adjustable air spring
$h_{0}\;\left(m\right)$	initial height of sprung mass	${\bar{h}}_{d}\;\left(m\right)$	maximum value of desired height
$b\;(N\cdot s\cdot m^{-1})$	damping coefficient of damper	$h_{max}\;\left(m\right)$	maximum value of sprung height
$p_{s}\;\left(\mathrm{Pa}\right)$	air spring pressure	d(t)	time-varying disturbances
$p_{0}\;\left(\mathrm{Pa}\right)$	initial air pressure	$d_{max}$	maximum value of disturbances
$v_{s}\;\left(m^{3}\right)$	air spring volume	$k_{t}\;\left(N\cdot m^{-1}\right)$	tire stiffness
$\dot{Q}\;\left(J\cdot s^{-1}\right)$	heat transfer rate	*u*	control input
$F_{s}$	air spring force	$F_{d}$	damping force

**Table 2:** Parameters for co-simulation.

Parameter	Value	Parameter	Value
$\bar{A}$	178 (mm${}^{2}$)	$k_{1}$	9
*b*	$1140\;(N\cdot s\cdot m^{-1})$	$k_{2}$	40
$h_{0}$	0.2047(m)	$k_{3}$	4
$\bar{h}$	0.4047(m)	$\lambda_{d}$	100
$m_{s}$	300(kg)	γ	1.4
$m_{u}$	30(kg)	$p_{0}$	5.11(Bar)
$d_{max}$	1.8	$p_{a}$	1.01(Bar)
$k_{t}$	$7.5\times 10^{6}\;\left(N\cdot m^{-1}\right)$	$b_{t}$	$300\;(N\cdot s\cdot m^{-1})$

**Table 3:** Performance index comparison of co-simulation.

Performance Index	Robust Controller in [14]	Proposed Controller	Improvement *
RMS of tracking error	$4.6824\times 10^{-1}$ (mm)	$1.6230\times 10^{-1}$ (mm)	65.3%
SD of tracking error	$4.6809\times 10^{-1}$ (mm)	$1.6012\times 10^{-1}$ (mm)	65.6%
Adjusting time	4 (s)	1 (s)	75%
* denotes relative to robust controller presented in [14].			

**Table 4:** Performance index comparison of co-simulation.

Performance Index	HMPC in [7]	Proposed Controller	Improvement *
RMS of tracking error	7.7785 (mm)	4.3781 (mm)	43.71%
SD of tracking error	6.0746 (mm)	3.2452 (mm)	46.58%
Adjusting time	3 (s)	1 (s)	66.67%
* denotes relative to HMPC presented in [7].			

## Data Availability

The data presented in this study are available on request from the corresponding author.
